# Herpes Simplex Virus Type 2 Immediate Early Protein ICP27 Inhibits IFN-β Production in Mucosal Epithelial Cells by Antagonizing IRF3 Activation

**DOI:** 10.3389/fimmu.2019.00290

**Published:** 2019-02-26

**Authors:** Xinmeng Guan, Mudan Zhang, Ming Fu, Sukun Luo, Qinxue Hu

**Affiliations:** ^1^State Key Laboratory of Virology, Wuhan Institute of Virology, Chinese Academy of Sciences, Wuhan, China; ^2^University of Chinese Academy of Sciences, Beijing, China; ^3^The Joint Center of Translational Precision Medicine, Guangzhou Institute of Pediatrics, Guangzhou Women and Children's Medical Center, Wuhan Institute of Virology, Chinese Academy of Science, Wuhan, China; ^4^Wuhan Children's Hospital (Wuhan Maternal and Child Healthcare Hospital), Tongji Medical College, Huazhong University of Science and Technology, Wuhan, China; ^5^Institute for Infection and Immunity, St George's University of London, London, United Kingdom

**Keywords:** HSV-2, ICP27, epithelial cells, IFN-β, IRF3

## Abstract

Herpes simplex virus type 2 (HSV-2) is the main cause of genital herpes and infections are common in the lower genital tract. Although neuronal and immune cells can be infected, epithelial cells, and keratinocytes are the primary HSV-2 target cells. HSV-2 establishes latency by evading the host immune system and its infection can also increase the risk of HIV-1 sexual transmission. Our pervious study found that HSV-2 immediate early protein ICP22, inhibited IFN-β production by interfering with the IRF3 pathway. However, ICP22-null HSV-2 did not completely lose the capability of suppressing IFN-β induction, suggesting the involvement of other viral components in the process. In this study, by using an *ex vivo* cervical explant model, we first demonstrated that HSV-2 can indeed inhibit IFN-β induction in human mucosal tissues. We further identified HSV-2 immediate early protein ICP27 as a potent IFN-β antagonist. ICP27 significantly suppresses the Sendai virus or polyinosinic-polycytidylic acid-induced IFN-β production in human mucosal epithelial cells, showing that ICP27 inhibits the IFN-β promoter activation, and IFN-β production at both mRNA and protein levels. Additional studies revealed that ICP27 directly associates with IRF3 and inhibits its phosphorylation and nuclear translocation, resulting in the inhibition of IFN-β induction. Our findings provide insights into the molecular mechanism underlying HSV-2 mucosal immune evasion, and information for the design of HSV-2 mucosal vaccines.

## Introduction

Herpes simplex virus type 2 (HSV-2) is a large dsDNA virus belonging to the α-Herpesviridae subfamily (1). HSV-2 is mainly transmitted by genital mucosa through sex, causing vesicles, and ulcers after acute infection and can be transported to dorsal root or cranial nerve ganglia to establish life-long latency (2, 3). According to a report by WHO, it is estimated that over 400 million people were infected with HSV-2 and 19.2 million new infections occurred worldwide in 2012 (4). Due to the greater and more fragile surface of the female reproductive tract, the risk of infection with HSV-2 in females is higher than that in males (5). Epidemiological studies have shown that HSV-2 infection can increase the risk of HIV-1 infection by 3–4 fold (6), with several mechanisms for this increased susceptibility being proposed. For instance, although HSV-2 can infect skin epithelial cells, immune cells and nerve cells, it initially infects mucosal epithelial cells during sexual transmission (7), which may facilitate HIV-1 transmission via perturbation of epithelial integrity. Due to the high positive-incidence of HSV-2 and common routes of transmission with HIV-1, HSV-2 mucosal infection and immune escape has attracted increased attention.

A virus infection initially activates innate immunity through recognition by host pattern recognition receptors (PRRs), including Toll-like receptors (TLRs), RIG-I-like receptors (RLR), and DNA sensors (8–10). These PRRs activate downstream signaling pathways using common components TBK-1 and IKK-ε, leading to the activation of IRF3 signaling (11). IRF3 is an important transcription factor which regulates the expression of type I interferons (IFNs) and IFN stimulate genes (ISGs). IRF3 exists as an inactive monomer in most cells. Upon activation, IRF3 is phosphorylated and assembled into dimers, and then translocated into the nucleus to initialize the transcription of IFN-β (12). Type I IFNs are normally expressed at low levels, and their expression can be enhanced through the JAK/STAT signaling pathway during viral or bacterial infections, resulting in the transcription activation of ISGs (13). The type I IFN family consists of IFN-α, IFN-β, IFN-ε, IFN-κ, and IFN-ω (14), with IFN-β being the most intensively investigated in antiviral innate immunity (15).

During HSV-2 infection, the induction of type I IFNs is extremely low (16), suggesting that HSV-2 has evolved strategies to antagonize IFN production. However, our current understanding of HSV-2 immune evasion is limited, whereas a large number of studies focusing on HSV-1 indicate the involvement of multiple countermeasures in subverting type I IFN production (17, 18). Given that most of these studies address how HSV-1 proteins interfere with IFN production or/and signaling using various cell lines as models (17), whether and how IFN induction is affected in the context of viral infection, at the tissue level, remain elusive. It is known that HSV-2 and HSV-1 exhibit substantial differences in latency and reactivation patterns (19), implying that they may use distinct mechanisms to counteract the host innate immunity. Previous studies by others indicated that HSV-2 virion host shutoff (vhs) protein UL41 suppresses IFN-β expression in human genital epithelial cells (20), while HSV-2 US2 activates NF-κB by binding to TAK1 (21). Our previous study revealed that HSV-2 immediate early protein (IE), ICP22 (US1), inhibits IFN-β production by antagonizing the association of IRF3 with the IFN-β promoter (22). Nevertheless, we observed that ICP22-null HSV-2 did not completely lose the inhibitory activity on IFN-β induction, while other IE proteins including ICP27 (UL54) also appeared to inhibit the activation of the IFN-β promoter, although the underlying mechanism remains to be fully addressed.

Our current study focused on whether and how HSV-2 and its IE protein ICP27, inhibit IFN-β production in mucosal epithelial cells. We found that HSV-2 can inhibit IFN-β induction in human cervical tissues. We further revealed that HSV-2 ICP27 significantly suppresses the Sendai virus or polyinosinic-polycytidylic acid-induced IFN-β production in human mucosal epithelial cells. Mechanistically, we demonstrated that HSV-2 ICP27 directly associates with IRF3 and inhibits its phosphorylation and nuclear translocation, resulting in the inhibition of IFN-β induction.

## Materials and Methods

### Cell Lines and Viruses

HEK 293T, HeLa, and ME180 cells were cultured in Dulbecco's modified Eagle medium (DMEM) (Hyclone), supplemented with 10% fetal bovine serum (FBS) (Gibico), 100 U/ml penicillin and 100 U/ml streptomycin (Genom). All cells were cultured at 37°C in a 5% CO_2_ incubator.

HSV-2 (G strain) was obtained from LGC standards and propagated in Vero cells. The Sendai virus (SeV) was propagated in embryonated eggs. Special pathogen-free embryonated eggs (Beijing Merial Vital Laboratory Animal Technology Corporation) were incubated at 37°C for 12 days before inoculation with 300 μl 100 HAU ml^−1^ SeV into the allantoic cavity of 12-day-old embryonated eggs and then incubated at 37°C for 72 h. SeV was collected from allantoic fluids and the titers were measured by hemagglutination (HA) assay using chicken red blood cells.

### Isolation of Primary Human Mucosal Epithelial Cells

All protocols involving human subjects were reviewed and approved by the local Research Ethics Committee of Wuhan Institute of Virology, Chinese Academy of Sciences. Informed written consents from the human subjects were obtained in this study, and informed written parental consents were obtained for all participants under the age of 16.

Human cervical or foreskin tissues were obtained from the Wuhan Children's Hospital (Wuhan Maternal and Child Healthcare Hospital), Tongji Medical College, Huazhong University of Science & Technology. Tissues were washed carefully with PBS and then minced into small pieces. Prepared tissue pieces were incubated with 5–10 ml Dispase II Solution (25 U/ml Dispase II in PBS pH 7.4 without Ca/Mg) containing penicillin and streptomycin. 10% FBS was then added to avoid excess damage to cells. Following an incubation at 4°C overnight, peeled off epidermis was rinsed with PBS, and placed into 3–5 ml 0.05% Trypsin with EDTA in a 50 ml conical tube. After an incubation in a 37°C water bath for 15–30 min with agitation every 5 min, twice the EDTA volume of medium with 10% FBS was added. The epidermal cells were released by inverting the tube several times or by pipetting the suspension. The cell/tissue solution was passed through a sterile sieve followed by centrifugation. Cell pellets were resuspended in EpiLife medium, and the isolated epithelial cells were cultured in 12-well plates at 37°C in a 5% CO_2_ incubator.

### Construction of Plasmids

Primers used for plasmid construction are listed in Supplementary Table 1. The open reading frame (ORF) of HSV-2 ICP27 was amplified from HSV-2 genomic DNA extracted from HSV-2 G strain by PCR. For some constructs, an N-terminal Flag or HA was introduced by PCR. PCR products were cloned into pcDNA3.1(+)/(-) (Invitrogen), and the constructed expression plasmids were named ICP27, ICP27-Flag, ICP27-HA, ICP27_(1−138aa)_, ICP27_(1−152aa)_, and ICP27_(1−302aa)_, respectively. All constructs were verified by DNA sequencing (Sunny Biotechnology). The reporter plasmid PRD(III-I)_4_-Luc was provided by Dr. Stephan Ludwig (University of Muenster, Muenster, Germany). The reporter plasmid p125-Luc and the internal control plasmid phRL-TK were described previously (23). IRF3 and IRF3-5D expression plasmids pIRES-hrGFP/IRF3-Flag and pIRES-hrGFP/IRF3-5D-Flag (constitutively active mutant of IRF3) were provided by Dr. Yiling Lin (Graduate Institute of Life Sciences, National Defense Medical Center, Taiwan, China). pEF-Flag-RIG-IN (a carboxy-terminally truncated, constitutively active RIG-I mutant) expression plasmid was provided by Dr. Takashi Fujita (Kyoto University, Kyoto, Japan). pcDNA3-MAVS-Flag expression plasmid was provided by Dr. Hanzhong Wang (Wuhan Institute of Virology, Wuhan, China). pcDNA3-TBK1-Flag and pcDNA3-IKKε-Flag expression plasmids were provided by Dr. Katherine Fitzgerald (University of Massachusetts Medical School, Worcester, MA). Plasmids encoding influenza virus PR8/NS1 and HSV-2 ICP22, respectively, were described previously (22).

### Dual Luciferase Report (DLR) Assay

HEK 293T cells were seeded in 24-well plates overnight and co-transfected with empty vector or plasmid encoding HSV-2 ICP27, truncated HSV-2 ICP27 or influenza virus NS1, reporter plasmid p125-Luc or PRD(III-I)_4_-Luc and internal control phRL-TK. Transfections were performed using Lipofectamine 2000 (Life Technology, 11668019) according to the manufacturer's instructions. At 24 h post-transfection, cells were stimulated with or without 100 HAU ml^−1^ SeV for 16 h. Cells were harvested and lysed, and the lysates were used for measuring firefly and Renilla luciferase activities using a Dual-luciferase Reporter Assay System (Promega, E1980) according to the manufacturer's instructions. For some experiments, HEK 293T cells were co-transfected with empty vector or ICP27 expression plasmid, reporter plasmid p125-Luc and internal control phRL-TK, together with plasmid encoding the IRF3 pathway inducer RIG-IN, MAVS, TBK-1, IKK-ε, or IRF3-5D. At 40 h post-transfection, the enzymatic activities of firefly and Renilla luciferase were measured.

### Immunoblot Assay

The proteins extracted from transfected or infected cells were prepared using Pierce™ IP Lysis Buffer (ThermoFisher Scientific, 87787) supplemented with protein inhibitor (cOmplete Protease Inhibitor Cocktail, 11697498001). The protein samples were resolved by SDS-PAGE and transferred onto PVDF membranes (0.45 μm, Millipore). Cytoplasmic and nuclear proteins were isolated using the Nuclear-Cytosol Extraction Kit (Applygen, P1200-50).

The antibody (Ab) anti-HSV-1 ICP27+HSV-2 ICP27 was purchased from Abcam (ab31631). Rabbit anti IRF3 Polyclonal Antibody, Rabbit anti PCNA Ab and Mouse mAb anti HA-tag were purchased from Proteintech (11312-1-AP, 10205-2-AP, and 66006-1-Ig). Rabbit mAb against phospho-IRF-3 (Ser396) was purchased from Cell Signaling Technology (4947S). Rabbit mAb against HA-tag and Mouse mAb against Flag-tag were purchased from Sigma-Aldrich (H6908 and F1804). Mouse mAb anti β-actin was purchased from Santa Cruz Biotechnology (sc81178). HRP-conjugated goat anti-rabbit IgG (H+L) and HRP-conjugated goat anti-mouse IgG (H+L) were purchased from ThermoFisher Scientific (ZB-2301 and ZB-2305). HRP-conjugated mouse anti-rabbit IgG (Light Chain) was purchased from Sangon Biotech (D110059-0100). Mouse IgG and Rabbit IgG were purchased from BOSTER (BA1046 and BA1045). Alexa Fluor 488-labeled Goat Anti-Mouse IgG (H+L), Alexa Fluor 647-labeled Goat Anti-Rabbit IgG (H+L) and DAPI Staining Solution were purchased from Beyotime (A0428, A0468, and C1006).

### RNA Isolation and Quantitative PCR

The transfected cells were collected and total RNAs were extracted using TRIzol (Invitrogen, 15596-026) according to the manufacturer's instructions. cDNA was synthesized by M-MLV Reverse Transcriptase (Promega, M1705). The newly synthesized cDNA was used as template for the amplification of *IFN-*β*, ISG15, ISG56, CXCL10*, and *GAPDH*. The primer pairs for *IFN-*β, *ISG15, ISG56, CXCL0*, and *GAPDH* were described previously (22, 24). Relative real-time quantitative PCR (RT-PCR) was performed on BioRad StepOne apparatus using a TransStart® Tip Green qPCR SuperMix (Transgen, AQ141-02), and GAPDH was used as an internal control with conditions of 95°C for 3 min, followed by 40 cycles of 95°C for 10 s, and 55°C for 30 s. The expression difference was calculated on the basis of 2^−ΔΔCt^ values.

### ICP27 Knockdown by siRNA

HSV-2 siRNA sequences were described previously (25), and are listed in the Supplementary Table 1. All siRNAs were synthesized by Eurofins Genomics. HeLa or ME180 cells were seeded in 6-well plates overnight. Negative control or siRNAs were transfected into HeLa or ME180 cells using Lipofectamine 2000 (Life Technology, 11668019) according to the manufacturer's instruction. At 4 h post-transfection, HeLa cells were infected with or without HSV-2 at an MOI of 1, or ME180 cells at an MOI of 0.5. At 20 h post-infection, cells were stimulated with or without 100 HAU ml^−1^ SeV for 16 h, and supernatants were harvested for ELISA or cells were lysed for DLR assay.

### Poly(I:C) Stimulation

HeLa or ME180 cells were seeded in 6-well plates overnight and transfected with empty vector, HSV-2 ICP27 expression plasmid, HSV-2 ICP22 expression plasmid or influenza virus NS1 expression plasmid. At 24 h post-transfection, cells were transfected with 2 μg/well Poly(I:C) (Sigma; P1530-25MG) using Lipofectamine 2000 (Life Technology, 11668019) or mock-transfected. At 16 h post-transfection, cells were lysed for DLR assay or supernatants were harvested for ELISA.

### ELISA for IFN-β

HEK 293T cells were seeded in 6-well plates overnight and transfected with empty vector, HSV-2 ICP27 expression plasmid or influenza virus NS1 expression plasmid. At 24 h post-transfection, cells were stimulated with or without 100 HAU ml^−1^ SeV for 16 h. Cell culture supernatants were collected and centrifuged to remove cell debris. Fifty microliters of supernatants were used for IFN-β detection using a VeriKine™ Human IFN Beta ELISA Kit (PBL Assay Science, 41410) according to the manufacturer's instructions.

### Immunofluorescence Assay

HeLa cells were seeded in 35 mm glass-bottom dishes and transfected with an empty vector, HSV-2 ICP27-HA expression plasmid or an influenza virus NS1 expression plasmid. At 24 h post-transfection, HeLa cells were stimulated with or without 100 HAU ml^−1^ SeV for 16 h. Cells were fixed with 4% paraformaldehyde and permeabilized with 0.2% Triton X-100. After three washes with PBS, cells were blocked with PBS containing 5% BSA for 1 h at room temperature, and then incubated with rabbit anti-human IRF3 polyclonal Ab and mouse anti HA-tag mAb at a dilution of 1:100 for 1 h at room temperature. After three washes with PBS, cells were incubated with Alexa Fluor 488-labeled Goat Anti-Mouse IgG (H+L) and Alexa Fluor 647-labeled Goat Anti-Rabbit IgG (H+L) at a dilution of 1:50 for 1 h at room temperature. Cells were subsequently washed and incubated with DAPI solution for 10 min at room temperature. Following the addition of 1 ml PBS into the dishes, cells were observed under a Multiphoton Confocal Microscope (Nikon, A1 MP STORM).

### Co-immunoprecipitation Assay

HEK 293T cells were seeded in 6-well plates and transfected with ICP27-Flag plasmid or empty vector. At 24 h post-transfection, cells were stimulated with or without 100 HAU ml^−1^ SeV for 16 h. The proteins extracted from transfected cells were prepared using Pierce™ IP Lysis Buffer (ThermoFisher Scientific, 87787). Three microgram mouse anti-Flag Ab or control mouse IgG was diluted in 200 μl PBS with 0.2% Tween-20 (PBST) and added to fresh Dynabeads protein G (Invitrogen, 10009D). After incubation with rotation at 4°C overnight, Dynabeads-Ab complexes were washed once with 200 μl PBST before mixed with the samples, followed by incubation at 4°C overnight. The complexes were washed three times with PBST, and target Ags were subjected to Western Blot analysis after elution by boiling.

### Binding Kinetic Analysis

HEK 293F cells were used for the expression and purification of ICP27-Flag and IRF3-Flag. For every 1 × 10^6^ cells, 1.5 μg expression plasmid was transfected into HEK 293F cells using Polyethylenimine (PEI) transfection reagent (Polysciences, 23966-1). Cells were cultured in FreeStyle 293 Expression Medium (Gibico, 12338018) at 37°C in a 5% CO_2_ incubator shaker at 135 rpm. At day 3 post-transfection, cells were harvested and lysed by ultrasonic treatment. The Flag-tagged protein was purified by Anti-DYKDDDDK G1 Affinity Resin (GeneScript, L00432) and eluted with 3 M NaCl. The purified protein was concentrated in PBS using 30 kDa Centrifugal Filter Units (Merck, UFC903008) for binding kinetic study.

The kinetics of binding was performed on a Forte-Bio Octet Red System as described previously (26). This system monitors interference of light reflected from the surface of a sensor to measure the thickness of molecules bound to the sensor surface. IRF3-Flag was conjugated with biotin at a molecular weight ratio of 1:3 at room temperature for 1 h followed by washes, and then concentrated in PBS to remove dissociative biotin. 10 microgram/milliliter biotinylated IRF3-Flag was coupled to Biosenors (Fortebio, 18-5019) and immersed in different concentration of ICP27-Flag (50, 200, 500, or 800 nM) for association and disassociation. The response in nm shift was recorded as a function of time.

### Statistical Analysis

All experiments were repeated at least three times and the data are presented as mean ± S.D., unless otherwise specified. Data analyses were performed with GraphPad Prism 7 software (GraphPad). A comparison between the two groups was analyzed using a two tailed unpaired Student's *t*-test. *P* < 0.05 was considered statistically significant.

## Results

### HSV-2 ICP27 Inhibits IFN-β Production in Human Mucosal Epithelial Cells

**Figure 1:**
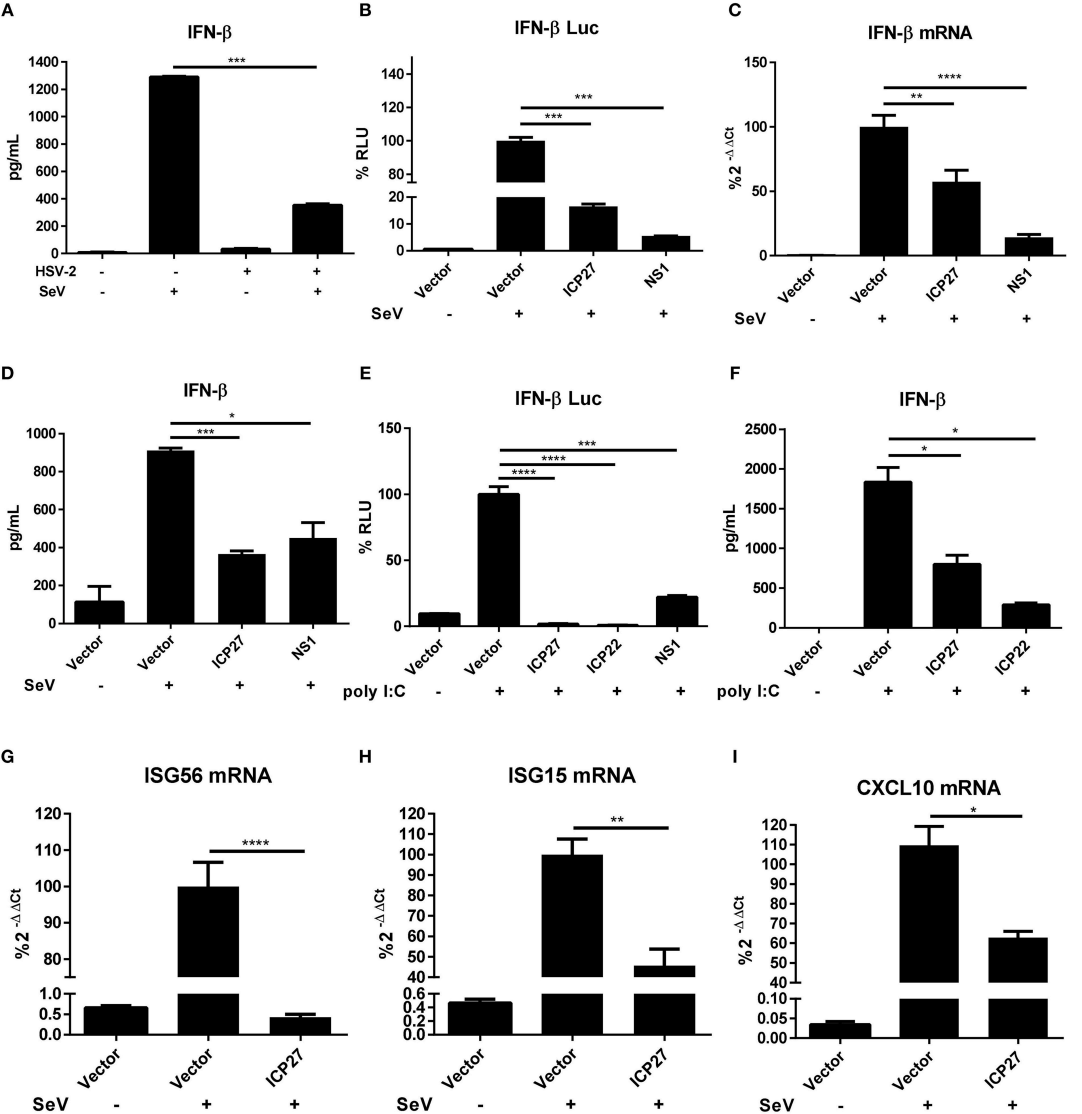
HSV-2 ICP27 inhibits IFN-β production in human mucosal epithelial cells. **(A)** HSV-2 inhibits IFN-β production in human cervical tissues. Human cervical tissues were prepared and infected with or without HSV-2. At 4 h.p.i, the tissues were stimulated with or without 100 HAU ml^−1^ SeV for 16 h, and the supernatants were harvested for ELISA. **(B)** HSV-2 ICP27 inhibits the activation of the IFN-β promoter. HEK 293T cells were seeded in 24-well plates and co-transfected with empty vector pcDNA3.1(+), HSV-2 ICP27 or influenza virus NS1 expressing plasmid, together with the reporter plasmid p125-Luc and the internal control phRL-TK. At 24 h post-transfection, cells were stimulated with or without 100 HAU ml^−1^ SeV for 16 h and lysed for DLR assay. **(C,D)** HSV-2 ICP27 inhibits IFN-β production at both mRNA and protein levels. HEK 293T cells were seeded in 6-well plates and transfected with empty vector pcDNA3.1(+), plasmid expressing HSV-2 ICP27 or influenza virus NS1. At 24 h post-transfection, cells were stimulated with or without 100 HAU ml^−1^ SeV for 16 h and total RNAs were extracted for RT-PCR **(C)**, while the supernatants were harvested for ELISA **(D)**. **(E)** HSV-2 inhibits the Poly(I:C)-induced activation of the IFN-β promoter. HeLa cells were seeded in 6-well plates and co-transfected with empty vector pcDNA3.1(+), plasmid expressing HSV-2 ICP27, ICP22 or the influenza virus NS1, together with the reporter plasmid p125-Luc and the internal control phRL-TK. At 24 h post-transfection, cells were transfected with 2 μg/well Poly(I:C) or mock-transfected for 16 h and lysed for DLR assay. **(F)** HSV-2 ICP27 inhibits Poly(I:C)-induced IFN-β production at protein level. HeLa cells were seeded in 6-well plates and transfected with empty vector pcDNA3.1(+), plasmid expressing HSV-2 ICP27 or ICP22. At 24 h post-transfection, cells were transfected with 2 μg/well Poly(I:C) or mock-transfected for 16 h, and the supernatants were harvested for ELISA. **(G–I)** HSV-2 ICP27 inhibits the production of ISGs. HEK 293T cells were seeded in 6-well plates and transfected with empty vector pcDNA3.1(+) or HSV-2 ICP27 expressing plasmid. At 24 h post-transfection, cells were stimulated with or without 100 HAU ml^−1^ SeV for 16 h and lysed for RT-PCR to measure the expression of *ISG56* **(D)**, *ISG15* **(E)**, and *CXCL10* **(F)** at the mRNA level. The data shown are representative of three independent experiments, with each condition performed in triplicate (mean ± SD) **(A–I)**. *P < 0.05; **P < 0.01; ***P < 0.001; ****P < 0.0001.

HSV-2 evades mucosal innate immunity, but the underlying mechanisms remain elusive (16). Our previous study showed that HSV-2 immediate early protein ICP22 strongly inhibited IFN-β production; however, knockout of ICP22 did not fully abolish the inhibitory activity on IFN-β production in the context of HSV-2 infection (22), while other IE proteins including ICP27 (UL54) also appeared to be involved, but the underlying mechanism remains to be fully investigated. In this study, we performed experiments to assess whether HSV-2 ICP27 indeed inhibits IFN-β induction, and if so, what is the underlying mechanism. We first confirmed that IFN-β was expressed at a very low level in HSV-2-infected human cervical tissues (Figure 1A), informing that HSV-2 inhibits IFN-β induction during mucosal infection. To assess the contribution of HSV-2 ICP27 in interfering with IFN-β induction, HEK 293T cells were seeded in 24-well plates overnight and co-transfected with empty vector pcDNA3.1(+), plasmid expressing HSV-2 ICP27 or the positive control influenza virus NS1 together with the reporter plasmid p125-Luc and the internal control phRL-TK. At 24 h post-transfection, cells were stimulated with or without 100 HAU ml^−1^ SeV for 16 h. As shown in Figure 1B, HSV-2 ICP27 strongly inhibited the activation of the IFN-β promoter. We subsequently examined whether HSV-2 ICP27 inhibits the production of IFN-β at mRNA or protein level. HEK 293T cells were seeded in 6-well plates overnight and transfected with pcDNA3.1(+), plasmid expressing HSV-2 ICP27 or the influenza virus NS1. At 24 h post-transfection, cells were stimulated with or without 100 HAU ml^−1^ SeV for 16 h. Total RNAs were extracted and IFN-β mRNA was analyzed by RT-PCR. As shown in Figure 1C, HSV-2 ICP27 significantly inhibited the production of IFN-β mRNA. The level of IFN-β proteins in the supernatants was measured by ELISA, showing that HSV-2 ICP27 significantly inhibited the production of INF-β at protein level (Figure 1D).

In addition to SeV stimulation, we also conducted experiments under the condition of the IFN-β expression induced by polyinosinic-polycytidylic acid [Poly(I:C)], an artificial dsRNA sequence which can stimulate RIG-I signaling pathway (27). HeLa cells were co-transfected with pcDNA3.1(+), plasmid expressing HSV-2 ICP27, HSV-2 ICP22, or the influenza virus NS1, together with the reporter plasmid p125-Luc and the internal control phRL-TK. At 24 h post-transfection, cells were stimulated with Poly(I:C) for 16 h and lysed for DLR assay. The results revealed that HSV-2 ICP27 strongly inhibited Poly(I:C)-induced activation of the IFN-β promoter (Figure 1E). In addition, HeLa cells were transfected with pcDNA3.1(+), plasmid expressing HSV-2 ICP27, HSV-2 ICP22, or the influenza virus NS1. At 24 h post-transfection, cells were stimulated with Poly(I:C) for 16 h, and supernatants were harvested for ELISA. The results revealed that HSV-2 ICP27 strongly inhibited Poly(I:C)-induced IFN-β induction at protein level (Figure 1F). In addition, HSV-2 ICP27 also significantly inhibited Poly(I:C) induced activation of the IFN-β promoter in ME180 cells (Supplementary Figure 1A).

Impaired expression of type I IFNs leads to reduced expression of the downstream interferons stimulated genes (ISGs) (28, 29). To examine the impact of HSV-2 ICP27 on ISG expression, HEK 293T cells were transfected with empty vector pcDNA3.1(+) or plasmid expressing HSV-2 ICP27, followed by stimulation with SeV for 16 h. Total RNAs were extracted, and the mRNAs of ISGs including *ISG56, ISG15*, and *CXCL10* were analyzed by RT-PCR. As shown in Figures 1G–I, HSV-2 ICP27 strongly inhibited the expression of *ISG56, ISG15*, and *CXCL10* at mRNA level.

**Figure 2:**
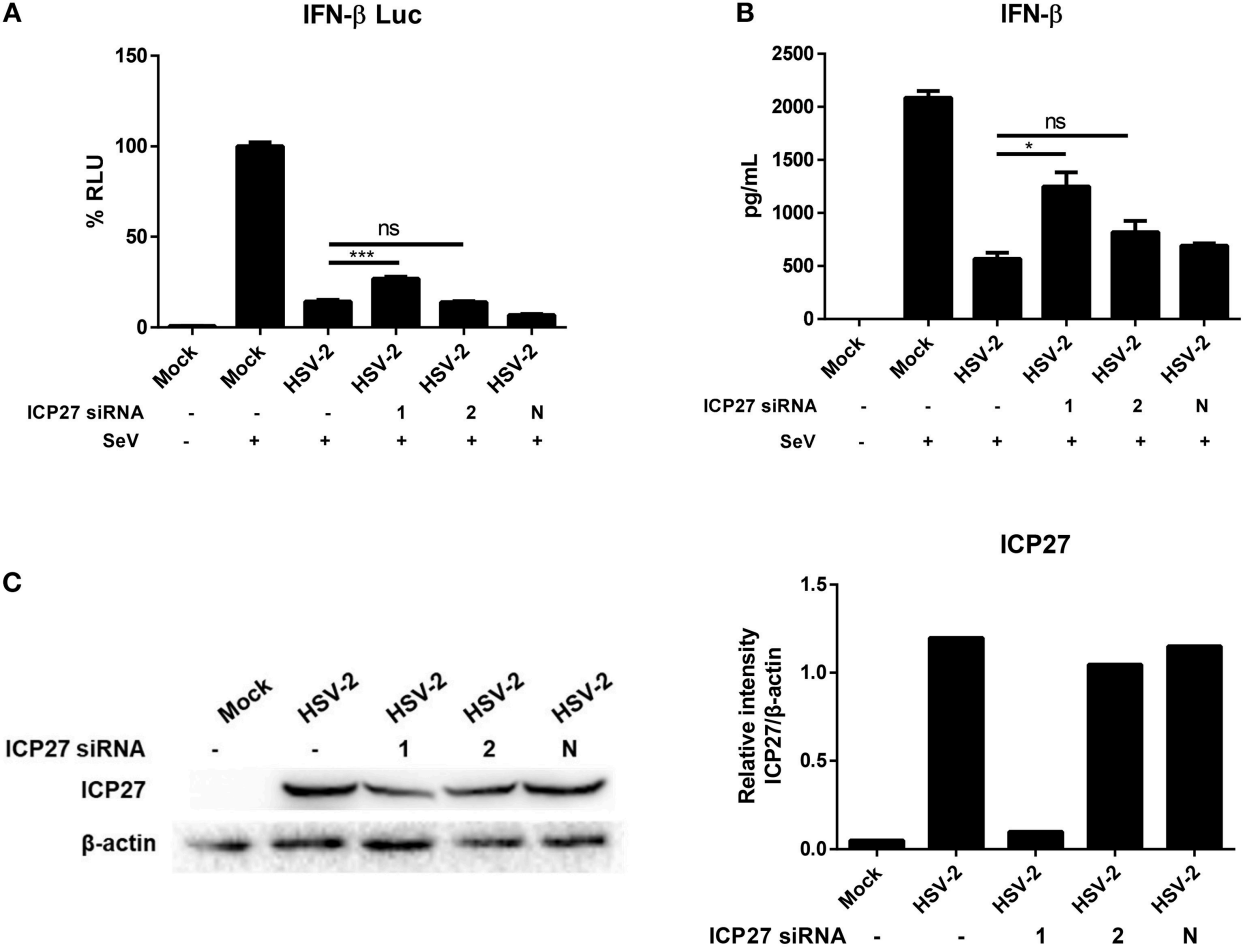
HSV-2 ICP27 inhibits IFN-β production in the context of virus infection. **(A)** Knockdown of ICP27 reduces the capability of HSV-2 in inhibiting the IFN-β promoter activation. HeLa cells were seeded in 6-well plates and co-transfected with HSV-2 ICP27 siRNA-1, siRNA-2, or negative control siRNA (N), together with the reporter plasmid p125-Luc and the internal control phRL-TK. At 4 h post-transfection, cells were infected with HSV-2 at an MOI of 1 or mock-infected. At 20 h.p.i, cells were stimulated with or without 100 HAU ml^−1^ SeV for 16 h and lysed for DLR assay. **(B)** Knockdown of ICP27 reduces the capability of HSV-2 in inhibiting the IFN-β production at the protein level. HeLa cells were seeded in 6-well plates and transfected with HSV-2 ICP27 siRNA-1, siRNA-2, or negative control siRNA (N). At 4 h post-transfection, cells were infected with HSV-2 at an MOI of 1 or mock-infected. At 20 h.p.i, cells were stimulated with or without 100 HAU ml^−1^ SeV for 16 h, and the supernatants were harvested for ELISA. **(C)** Knockdown efficiency of HSV-2 ICP27 siRNA. HeLa cells were seeded in 6-well plates and transfected with HSV-2 ICP27 siRNA-1, siRNA-2, or negative control siRNA (N). At 4 h post-transfection, cells were infected with HSV-2 at an MOI of 1 or mock-infected. At 20 h.p.i, cells were stimulated with or without 100 HAU ml^−1^ SeV for 16 h and lysed for Western Blot. Gray scale scanning was performed by Image J software (version 1.52a). The data shown are representative of three independent experiments, with each condition performed in triplicate (mean ± SD) **(A,B)**. *P < 0.05; ***P < 0.001. One representative experiment out of three is shown **(C)**.

Having demonstrated the critical role of HSV-2 ICP27 in inhibiting IFN-β induction, we next conducted experiments to examine the impact of HSV-2 ICP27 on IFN-β induction in the context of HSV-2 infection. HeLa cells were co-transfected with the reporter plasmid p125-Luc and the internal control phRL-TK, together with negative control siRNA, ICP27 siRNA-1 or ICP27 siRNA-2, followed by infection with HSV-2. Cells were then stimulated with or without 100 HAU ml^−1^ SeV for 16 h and lysed for DLR assay. As shown in Figure 2A, knockdown of HSV-2 ICP27 by ICP27 siRNA-1 reduced the capability of HSV-2 in inhibiting IFN-β production at promoter activation level. To assess the impact of HSV-2 ICP27 on IFN-β production at protein level, HeLa cells were treated with negative control siRNA, ICP27 siRNA-1 or ICP27 siRNA-2, followed by infection with HSV-2. At 24 h post-infection, supernatants were collected for ELISA and cells were lysed for Western Blot. As shown in Figure 2B, knockdown of HSV-2 ICP27 by ICP27 siRNA-1 reduced the capability of HSV-2 in inhibiting IFN-β production at the protein level. Knockdown of HSV-2 ICP27 was detected by a monoclonal antibody against HSV-2 ICP27, while β-actin was used as an internal control (Figure 2C). We also found that knockdown of HSV-2 ICP27 reduced the capability of HSV-2 in inhibiting IFN-β promoter activation in ME180 cells (Supplementary Figure 1B).

**Figure 3:**
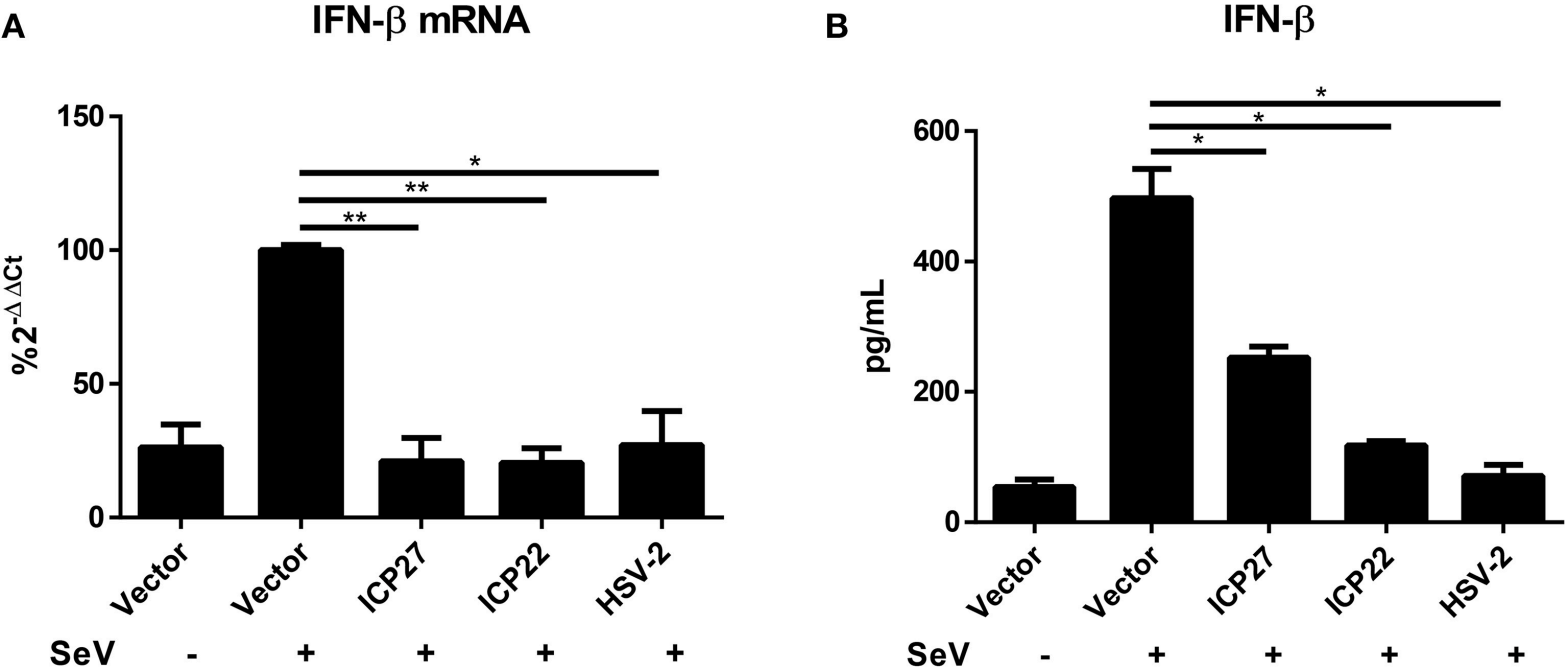
HSV-2 ICP27 inhibits IFN-β production in primary mucosal epithelial cells. **(A,B)** HSV-2 ICP27 inhibits IFN-β production in human foreskin epithelial cells. Primary epithelial cells were isolated from human foreskin tissues and transfected with empty vector pcDNA3.1(+), plasmid expressing HSV-2 ICP27 or ICP22 or infected with HSV-2. At 24 h post-transfection, cells were stimulated with or without 100 HAU ml^−1^ SeV for 16 h, and total RNAs were extracted for RT-PCR **(A)**, while the supernatants were harvested for ELISA **(B)**. The data shown are representative of three independent experiments, with each condition performed in triplicate (mean ± SD) **(A,B)**. *P < 0.05; **P < 0.01.

HSV-2 mainly infects epithelial cells and causes genital herpes. In addition to human cervical tissue and cervicovaginal epithelial cell lines, we performed experiments using primary human foreskin epithelial cells. Human foreskin epithelial cells were isolated and transfected with empty vector pcDNA3.1(+), plasmid expressing HSV-2 ICP27 or ICP22, or infected with HSV-2, followed by stimulation with or without 100 HAU ml^−1^ SeV for 16 h. Total RNAs were extracted for RT-PCR and supernatants were collected for ELISA. In primary human foreskin epithelial cells, HSV-2 ICP27 significantly inhibited SeV-induced IFN-β production at both mRNA (Figure 3A) and protein (Figure 3B) levels. These results indicated that HSV-2 ICP27 can inhibit IFN-β induction in primary mucosal epithelial cells.

Altogether, the above findings inform that HSV-2 ICP27 plays an essential role in interfering with the induction of IFN-β in human cervical tissues, cervicovaginal epithelial cell lines, and primary human mucosal epithelial cells.

### HSV-2 ICP27 Inhibits IFN-β Production Through IRF3 Signaling Pathway

**Figure 4:**
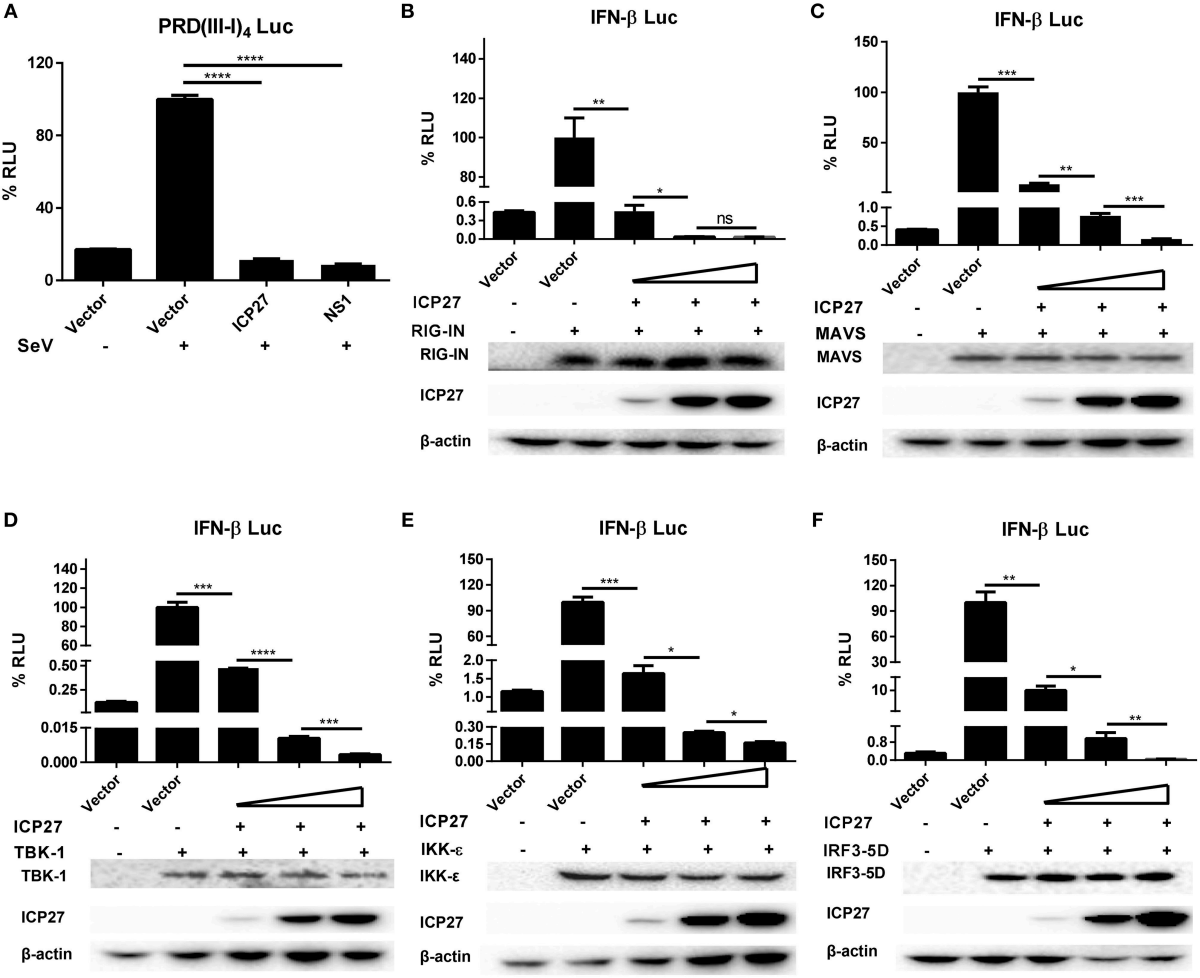
HSV-2 ICP27 inhibits IFN-β production through IRF3 signaling pathway. **(A)** HSV-2 ICP27 inhibits the IRF3 promoter element PRD(III-I)_4._ HEK 293T cells were seeded in 24-well plates and co-transfected with empty vector pcDNA3.1(+), plasmid expressing HSV-2 ICP27 or influenza virus NS1, together with PRD(III-I)_4_-Luc and the internal control phRL-TK. At 24 h post-transfection, cells were stimulated with or without 100 HAU ml^−1^ SeV for 16 h and lysed for DLR assay. **(B–F)** HSV-2 ICP27 inhibits RIG-I, MAVS, TBK1, IKK-ε, or IRF3-5D induced IFN-β promoter activation in a dose-dependent manner. HEK 293T cells were seeded in 24-well plates and co-transfected with empty vector pcDNA3.1(+) or HSV-2 ICP27 expressing plasmid, and plasmid expressing IRF3 signaling pathway component RIG-IN, MAVS, TBK1, IKK-ε, or IRF3-5D, together with the reporter plasmid p125-Luc and the internal control phRL-TK. At 40 h post-transfection, cells were lysed for DLR assay. At the same time, cells were lysed for Western Blot. RIG-I, MAVS, TBK1, IKK-ε, and IRF3-5D were detected by Anti-FLAG Ab while actin was detected by Anti-actin Ab. The data shown are representative of three independent experiments, with each condition performed in triplicate (mean ± SD) **(A–F)**. *P < 0.05; **P < 0.01; ***P < 0.001; ****P < 0.0001. For Western Blot, one representative experiment out of three is shown **(B–F)**.

HSV-1 ICP27 has recently been reported to inhibit type I IFNs by interacting with STING-TBK1 complex in macrophages (30), whereas our previous study found that HSV-2 can interrupts RIG-I mediated IFN-β signaling pathway in human epithelial cells (22). Given that DNA viruses produce dsRNAs during viral replication, which can be recognized by RNA sensors like RIG-I (31, 32), our current study focused on how HSV-2 ICP27 interferes with dsRNA-mediated induction of type I IFNs. We first determined whether HSV-2 ICP27 affects the IRF3-mediated signaling pathway. HEK 293T cells were seeded in 24-well plates overnight and co-transfected with empty vector pcDNA3.1(+), plasmid expressing HSV-2 ICP27 or the influenza virus NS1, together with PRD(III-I)_4_-Luc which contains four repeats of IRF3 responsive domain of the IFN-β promoter and the internal control phRL-TK. At 24 h post-transfection, cells were stimulated with or without 100 HAU ml^−1^ SeV for 16 h and lysed for DLR assay. As shown in Figure 4A, HSV-2 ICP27 blocks the activation of the IRF3 responsive promoter induced by SeV.

We next carried out experiments to address whether HSV-2 ICP27 directly affects the IRF3 signaling pathway. HEK 293T cells were seeded in 24-well plates overnight and co-transfected with a plasmid expressing IRF3 signaling pathway component RIG-IN, MAVS, TBK1, IKK-ε, or IRF3-5D and HSV-2 ICP27 expressing plasmid or empty vector pcDNA3.1(+), together with the reporter plasmid p125-Luc and the internal control phRL-TK. As shown in Figures 4B–F, HSV-2 ICP27 blocks the activation of the IFN-β promoter induced by all the tested IRF3 signaling pathway components in a dose-dependent manner.

Altogether, the above results indicate that, unlike HSV-1 ICP27, HSV-2 ICP27 inhibits IFN-β induction through an IRF3 dependent pathway.

### HSV-2 ICP27 Blocks IRF3 Activation by Physically Interacting With IRF3

**Figure 5:**
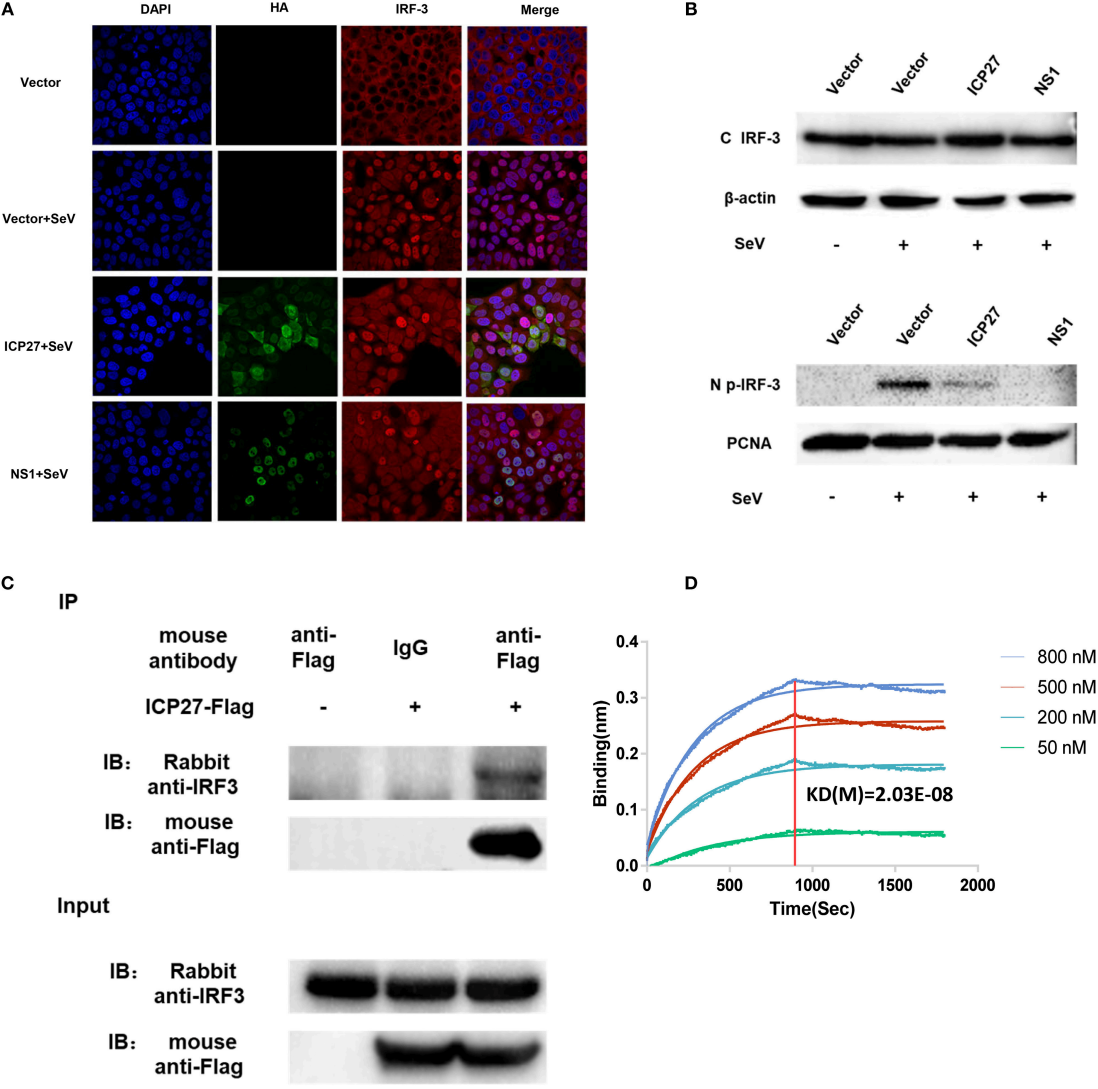
HSV-2 ICP27 blocks IRF3 activation by physical interaction. **(A)** HSV-2 ICP27 interferes with the nuclear translocation of IRF3. HeLa cells were seeded in 35 mm glass-bottom dishes and transfected with empty vector pcDNA3.1(+), plasmid expressing HSV-2 ICP27-HA or influenza virus NS1-HA. At 24 h post-transfection, cells were stimulated with or without 100 HAU ml^−1^ SeV for 16 h and prepared for immunofluorescence assay. IRF3 was detected under 647 nm wavelength, ICP27-HA or NS1-HA under 488 nm wavelength, and nuclei under 405 nm wavelength. **(B)** HSV-2 ICP27 inhibits IRF3 phosphorylation. HEK 293T cells were seeded in 6-well plates and transfected with pcDNA3.1(+), plasmid expressing HSV-2 ICP27 or influenza virus NS1. At 24 h post-transfection, cells were stimulated with or without 100 HAU ml^−1^ SeV for 16 h. Nuclear and cytoplasmic proteins were isolated, and phosphorylated IRF3 was detected with a p-IRF3 mAb. **(C)** HSV-2 ICP27 interacts with endogenous IRF3. HEK 293T cells were seeded in 6-well plates and transfected with pcDNA3.1(+) or HSV-2 ICP27-Flag. At 24 h post-transfection, cells were stimulated with or without 100 HAU ml^−1^ SeV for 16 h and lysed for immunoprecipitation assay. One representative experiment out of three is shown **(A–C)**. **(D)** Kinetics of ICP27-IRF3 binding. The kinetics of binding was performed on a Forte-Bio Octet Red System. Purified IRF3 was conjugated with biotin at a molecular weight ratio of 1:3. Ten microgram/milliliter biotinylated IRF3-Flag was coupled to Biosenors and immersed in different concentration of ICP27-Flag (50, 200, 500, or 800 nM) for association and disassociation. The response in nm shift was recorded as a function of time. KD (M) = 2.03E-08.

There are two main steps involved in IRF3 activation, phosphorylation and nuclear translocation (12). We first investigated whether HSV-2 ICP27 interferes with IRF3 nuclear translocation. Hela cells were seeded in 35 mm glass-bottom dishes overnight and transfected with plasmid expressing HSV-2 ICP27-HA or influenza virus NS1, or empty vector pcDNA3.1(+). At 24 h post-transfection, cells were stimulated with or without 100 HAU ml^−1^ SeV for 16 h. Indirect immunofluorescence assay was performed to assess IRF3 localization in the presence or absence of HSV-2 ICP27. As shown in Figure 5A, IRF3 translocation from the cytoplasm to the nucleus was partially blocked in the presence of HSV-2 ICP27.

Subsequent experiments were conducted to examine whether HSV-2 ICP27 blocks the phosphorylation of IRF3. HEK 293T cells were seeded in 6-well plates overnight and transfected with plasmid expressing HSV-2 ICP27 or influenza virus NS1, or empty vector. At 24 h post-transfection, cells were stimulated with or without 100 HAU ml^−1^ SeV for 16 h. The phosphorylation of IRF3 was detected by an anti-p-IRF3 Ab, showing that HSV-2 ICP27 significantly inhibited IRF3 phosphorylation in cells (Supplementary Figure 2), and particularly in the nucleus (Figure 5B).

We next asked whether there is an interaction between HSV-2 ICP27 and IRF3. Co-immunoprecipitation (Co-IP) was therefore carried out. HEK 293T cells were seeded in 6-well plates overnight and transfected with plasmid expressing HSV-2 ICP27-Flag or empty vector pcDNA3.1(+). At 24 h post-transfection, cells were stimulated with or without 100 HAU ml^−1^ SeV for 16 h, followed by Co-IP with a control IgG or an anti-Flag Ab. The precipitates were analyzed by Western Blot using an anti-IRF3 Ab against endogenous IRF3. As shown in Figure 5C, HSV-2 ICP27 was able to specifically precipitate the endogenous IRF3, indicating a physical interaction between HSV-2 ICP27 and IRF3. To further confirm the interaction between IRF3 and ICP27, we purified ICP27 and IRF3 proteins, and carried out binding kinetic analyses. A biotinylated IRF3-Flag was coupled to Biosenors and immersed in different concentrations of the ICP27-Flag for association and disassociation. As shown in Figure 5D, there was a strong association between IRF3 and ICP27, and a higher concentration of ICP27 resulted in a stronger association.

Altogether these results indicate that HSV-2 ICP27 antagonizes the IRF3 signaling pathway by interacting with IRF3.

### The 1-138aa Domain of HSV-2 ICP27 Is Mainly Responsible for the Inhibition of IFN-β Induction

**Figure 6:**
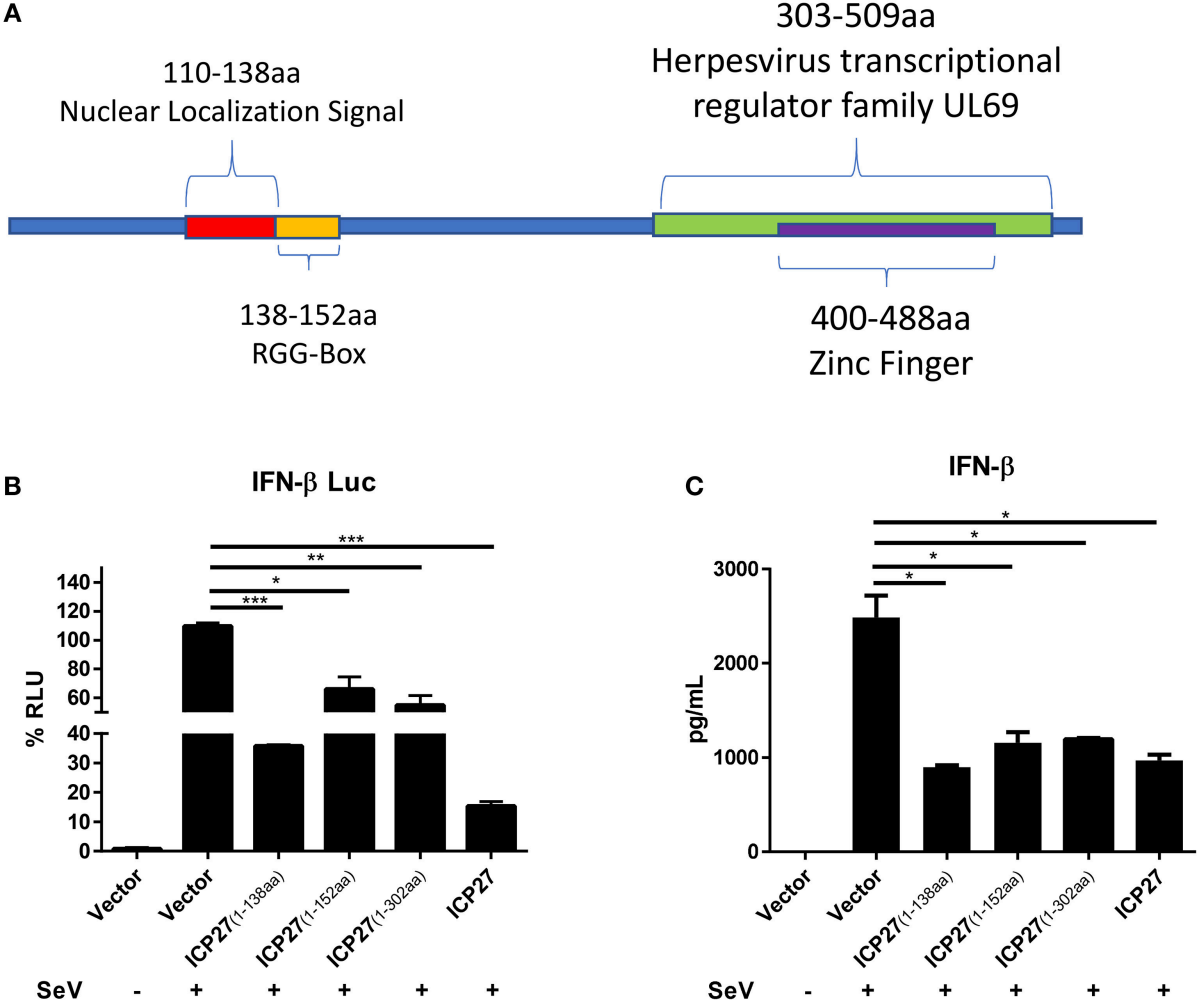
Mapping the key domain of HSV-2 ICP27 that inhibits IFN-β production. **(A)** Illustration of HSV-2 ICP27 structural domain. 110-138aa is the nuclear localization signal of ICP27; 138-152aa is the RGG-box domain; 303-509aa is the Herpesvirus transcriptional regulator family UL69; 400-488aa is the zinc ring finger domain. **(B)** The 1-138aa domain of ICP27 inhibits the IFN-β promoter activation. HEK 293T cells were seeded in 24-well plates and co-transfected with empty vector pcDNA3.1(+), plasmid expressing HSV-2 ICP27 or its truncation mutant, together with the reporter plasmid p125-Luc and the internal control phRL-TK. At 24 h post-transfection, cells were stimulated with or without 100 HAU ml^−1^ SeV for 16 h and lysed for DLR assay. **(C)** The 1-138aa domain of ICP27 inhibits IFN-β induction at protein level. HEK 293T cells were seeded in 24-well plates and transfected with empty vector pcDNA3.1(+), plasmid expressing HSV-2 ICP27 or its truncation mutant. At 24 h post-transfection, cells were stimulated with or without 100 HAU ml^−1^ SeV for 16 h, and supernatants were harvested for ELISA. The data shown are representative of three independent experiments, with each condition performed in triplicate (mean ± SD) **(A,B)**. *P < 0.05; **P < 0.01; ***P < 0.001.

To map the functional region of HSV-2 ICP27 involved in the inhibition of IFN-β production, we constructed several HSV-2 ICP27 truncation mutants according to its structure (https://www.uniprot.org/uniprot/P28276) (Figure 6A). HEK 293T cells were co-transfected with empty vector pcDNA3.1(+), truncated or full-length HSV-2 ICP27 expressing plasmid, with the reporter plasmid p125-Luc and the internal control phRL-TK. At 24 h post-transfection, cells were stimulated with or without 100 HAU ml^−1^ SeV for 16 h and lysed for DLR assay. To measure IFN-β at protein level, HEK 293T cells were transfected with empty vector pcDNA3.1(+), truncated or full-length HSV-2 ICP27 expressing plasmid, followed by stimulation with or without 100 HAU ml^−1^ SeV for 16 h, and supernatants were harvested for ELISA. As shown in Figures 6B,C, the 1-138aa domain of HSV-2 ICP27 significantly inhibited IFN-β induction at both promoter activation and protein levels, and the capability was comparable to that of the full-length HSV-2 ICP27. The expression of ICP27 mutants was confirmed by Western Blot (Supplementary Figure 3). In contrast to that of HSV-1 ICP27 (30), we did not observe a direct contribution of the RGG box, which is located in the 138-152aa domain of HSV-2 ICP27, to the inhibited IFN-β induction. Our findings indicate that the 1-138aa domain, rather than the RGG box of HSV-2 ICP27 is mainly responsible for the inhibition of IFN-β induction.

## Discussion

HSV-2 is a large enveloped dsDNA virus, and its infections are known to be restricted to mucosal and keratinized epithelia and neuronal ganglia. HSV-2 infection causes genital herpes with sexual transmission being the main route. It is known that HSV-2 can evade the host mucosal innate immunity, but the underlying mechanisms remain to be defined (16). Our current study has demonstrated that HSV-2 immediate early protein, ICP27, interferes with RIG-I-MAVS-IRF3-mediated IFN-β induction in mucosal epithelial cells and HEK 293T cells. Mechanistically, ICP27 directly associates with IRF3 and inhibits its phosphorylation and nuclear translocation, resulting in the inhibition of IFN-β induction. Findings in this study reveal an unconventional strategy exploited by a dsDNA virus to interrupt the type I IFN signaling pathway.

It is generally accepted that DNA viruses are recognized by DNA sensors such as TLR9 and cGAS (33), while RNA viruses are sensed by RNA sensors including TLR3, TLR7/8, RIG-I and MDA5 (34, 35). Indeed, a number of studies on HSV-1 reported that the virus can interfere with the cGAS-STING pathway to inhibit type I IFN induction (30, 36–38). Of interest, we previously found that HSV-2 can also interrupt RIG-I-MAVS-IFN-β pathway in human mucosal epithelial cells (22). One explanation is that, DNA viruses produce dsRNAs during their replication cycles, which can be recognized by RNA sensors like RIG-I (31, 32). In addition, STING has also been reported to be involved in RNA virus recognition (39). In the case of HSV-2, its RNAs are rapidly generated during the life cycle of its primary infection (40), which may represent an important alternative source of pathogen-associated molecular patterns to trigger innate immune responses.

HSV-2 and HSV-1 have different initial infection and transmission sites, with HSV-2 mainly resulting in genital infections and HSV-1 normally causing orofacial infections (41). In addition, HSV-2 has a high positive-incidence of infections and common mucosal transmission routes with HIV-1 (42). For instance, HSV-2 predominantly infects mucosal epithelial cells which forms the primary mucosal barriers against HIV-1 infection, and interruption of these barriers may facilitate HIV-1 transmission (5). In the current study, we found that HSV-2 can significantly inhibit IFN-β induction in human cervical tissues. Moreover, by using mucosal epithelial cell lines and primary mucosal epithelial cells as models, we demonstrated that HSV-2 ICP27 contributes to such inhibited IFN-β induction. The significance of HSV-2 ICP27 in inhibiting IFN-β induction was further confirmed in the context of virus infection by the specific siRNA knockdown of HSV-2 ICP27, although knockdown of HSV-2 ICP27 did not fully abolish HSV-2-mediated inhibition of IFN-β induction. Given the complexity of the HSV-2 genome encoding at least 74 proteins, it is highly likely that other unidentified HSV-2 proteins may also contribute to the suppression of IFN-β production. Because of the importance of IFN-β in inhibiting SHIV mucosal transmission (43), future studies are warranted to investigate whether HSV-2 infection-mediated IFN-β reduction plays a role in enhancing HIV-1 genital transmission.

We revealed in the current study that HSV-2 ICP27 can inhibit IFN-β production via the RIG-I-MAVS pathway. HSV-1/2 ICP27 is an essential multifunctional immediate early protein which regulates viral gene expression (44, 45). To date, most of the studies on ICP27 have focused on HSV-1 (44–46). For instance, HSV-1 ICP27 was shown to inhibit the phosphorylation and accumulation of STAT-1 in the nucleus, resulting the interruption of type I IFN signaling (47). In agreement, HSV-1 ICP27 knockout enhanced the activation of IRF3 and NF-κB in macrophages and DCs (48). More recently, HSV-1 ICP27 has been reported to inhibit type I IFN induction by interfering with the cGAS–STING–TBK1 signaling pathway in human macrophages (30). However, HSV-1 ICP27 appeared not to interfere with TBK1 phosphorylation, and the association of HSV-1 ICP27 with TBK1 required STING (30). In agreement, we found that, HSV-2 ICP27 strongly inhibited the production of IFN-β in HEK 293T cell line (22), which does not express STING (49), further strengthening that HSV-2 ICP27 can inhibit IFN production through a cGAS–STING–TBK1 independent pathway.

IRF3 plays a crucial role in type I IFN-mediated antiviral immune response (50). Activation of IRF3 during IFN-β production has several key steps: phosphorylation, dimerization, and cytoplasm-to-nucleus translocation. We previously found that, HSV-2 ICP22 inhibits IFN-β induction by antagonizing the association of IRF3 with the IFN-β promoter without suppressing the phosphorylation and nuclear translocation of IRF3 (22). Unlike the mechanism used by HSV-2 ICP22, we demonstrated in the current study that HSV-2 ICP27 interacts with IRF3 and interferes with IRF3 activation by blocking IRF3 phosphorylation and nuclear translocation, thereby inhibiting the production of IFN-β and ISGs. Given the complexity of HSV-2 genome containing over 70 genes, it is probable that multiple HSV-2 components likely contribute to the suppression of type I IFN induction by HSV-2. Indeed, although our current understanding of HSV-2 immune evasion is limited, work on HSV-1 has revealed multiple countermeasures in subverting type I IFN production (17, 18). By designing and analyzing HSV-2 ICP27 truncation mutants, we found that the 1-138aa region of HSV-2 ICP27 is the key functional domain responsible for HSV-2 ICP27-mediated IFN-β reduction. In contrast, a study on HSV-1 ICP27 has shown that its RGG box, which is located in the region of 139-152aa, is the main determinant antagonizing the cytosolic DNA-stimulated IFN-β production, by targeting TBK1 and STING (30). Although HSV-2 ICP27 shares 79% of amino acid sequence with HSV-1 ICP27, the key function domain, the N-terminus is only 65% identical, which may explain the differences in biological functions. For instance, compared with HSV-1 ICP27, HSV-2 ICP27 is less efficient in promoting the cytoplasmic localization of ICP4, another important immediate early protein of HSV 1/2 (44). In addition, the capability of HSV-2 ICP27 in inhibiting IFN-β production appeared to be much stronger than that of HSV-1 ICP27 when the ICP27 of HSV-1 was replaced with HSV-2 ICP27 (30), indicating the distinction of HSV-2 ICP27 in inhibiting IFN-β production.

**Figure 7:**
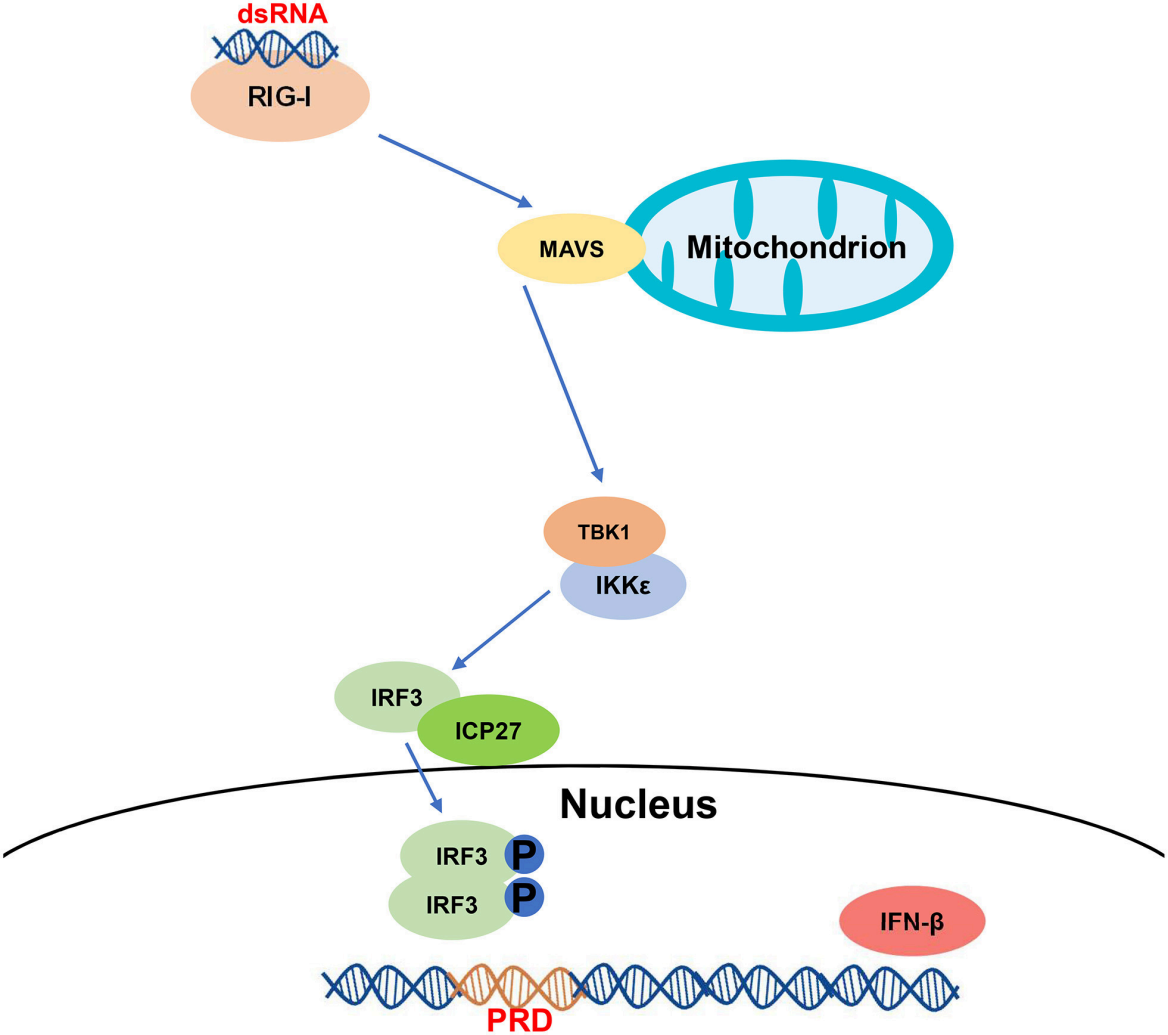
A schematic model of the mechanism by which HSV-2 ICP27 inhibits IFN-β production. HSV-2 dsRNA is detected by RIG-I, which recruits MAVS and downstream TBK1 and IKK-ε complex. HSV-2 ICP27 interacts with IRF3 and interferes with IRF3 activation by blocking IRF3 phosphorylation and nuclear translocation, thereby inhibiting the production of IFN-β and ISGs.

In conclusion, we have demonstrated that HSV-2 ICP27 inhibits IFN-β induction in human cervical tissues, cervicovaginal epithelial cell lines, and primary human mucosal epithelial cells. We further addressed the underlying mechanism and proposed one model based on the RIG-I-MAVS-IRF3 pathway (Figure 7). During HSV-2 infection of mucosal epithelial cells, a number of by-products, such as viral dsRNA, are yielded and can be recognized by the RIG-I receptor. RIG-I binds to dsRNA through the helicase domain and signals through caspase activation and recruitment domains to the adaptor MAVS. Engagement of RIG-I initiates signaling through two downstream protein kinase complexes, TBK-1/IKK-ε, leading to the phosphorylation and dimerization of IRF3. IRF3 dimers translocate from the cytoplasm into the nucleus to bind to the IFN-β promoter, and promote IFN-β transcription (51). On the other hand, HSV-2 immediate early protein ICP27 interacts with IRF3, and interferes with IRF3 phosphorylation and nuclear translocation, thereby inhibiting the production of IFN-β and ISGs. Our findings provide important information for understanding how HSV-2 evades mucosal innate immunity and a potential viral target for intervention.

## Author Contributions

QH conceived the study. XG, MZ, and MF conducted the experiments. SL provided tissues samples. XG, MZ, and QH analyzed the data. XG and QH wrote the manuscript. All authors reviewed the manuscript.

### Conflict of Interest Statement

The authors declare that the research was conducted in the absence of any commercial or financial relationships that could be construed as a potential conflict of interest.

## Abbreviations

HSV-2: herpes simplex virus type 2
SeV: Sendai virus
Poly(I:C): polyinosinic-polycytidylic acid
PEI: Polyethylenimine
DLR: dual luciferase report
Co-IP: co-immunoprecipitation
RT-PCR: real-time quantitative PCR
PRR: pattern recognition receptor
TLR: Toll-like receptor
RLR: RIG-I-like receptor
ISG: IFN stimulate gene
FBS: fetal bovine serum
DMEM: Dulbecco's modified Eagle medium
ORF: open reading frame.
