# Supplementary material for: Herpes Simplex Virus Type 2 Immediate Early Protein ICP27 Inhibits IFN-β Production in Mucosal Epithelial Cells by Antagonizing IRF3 Activation
Source: Front Immunol. 2019 Feb 26;10:290. doi: 10.3389/fimmu.2019.00290 (PMC6399465; doi:10.3389/fimmu.2019.00290)
Supplement: Supplementary file 1 [file Table_1.doc]

Supplementary Material

**Herpes simplex virus type 2 immediate early protein ICP27 inhibits IFN-β production in mucosal epithelial cells by antagonizing IRF3 activation**

**Xinmeng Guan^1,2^, Mudan Zhang^3^, Ming Fu^1,2^, Sukun Luo^4^, Qinxue Hu^1,5^**

^1^ State Key Laboratory of Virology, Wuhan Institute of Virology, Chinese Academy of Sciences, Wuhan, China

^2^ University of Chinese Academy of Sciences, Beijing, China

^3^The Joint Center of Translational Precision Medicine, Guangzhou Institute of Pediatrics, Guangzhou Women and Children’s Medical Center, Wuhan Institute of Virology, Chinese Academy of Science, Wuhan, China,

^4^Wuhan Children's Hospital (Wuhan Maternal and Child Healthcare Hospital, Tongji Medical College, Huazhong University of Science & Technology, Wuhan, China

^5^Institute for Infection and Immunity, St George’s University of London, London, UK

*** Correspondence:**Dr. Qinxue Hu: [qhu@wh.iov.cn](mailto:qhu@wh.iov.cn); Dr. Mudan Zhang: [mudan@wh.iov.cn](mailto:mudan@wh.iov.cn)

# Supplementary Table.1. Primers used in the study

| ICP27-Flag-F | GGCCAAGCTTATGGATTACAAGGATGACGACGATAAGGCTACCGACATTGATATGCT |
| --- | --- |
| ICP27-HA-F | GAATTAGGATCCATGTACCCATACGACGTCCCAGACTACGCTGCTACCGACATTGATA |
| ICP27-Flag-F | GGCCAAGCTTATGGATTACAAGGATGACGACGATAAGGCTACCGACATTGATATGCT |
| ICP27_1-138aa_-R | TATAGGATCCCTATCGGGGATGCGGTGCCTTG |
| ICP27_1-152aa_-R | AATTAAGGATCCCTAGCCGTATCGACCCCGGCCCC |
| ICP27_1-302aa_-R | TTAAGGATCCCTACCCCCCCGCTTGGGTGG |
| ICP27-R | TATAGGATCCCTAAAATAGGGAGTTGCAGTAGAA |
| *ifnb*-F | CAAATTGCTCTCCTGTTGTGCTTC |
| *ifnb*-R | AATGCGGCGTCCTCCTTCT |
| *gapdh*-F | GGGAAGCTCACTGGCATGG |
| *gapdh*-R | TTACTCCTTGGAGGCCATGT |
| *ifit1-*F | CCTCCTTGGGTTCGTCTACA |
| *ifit1-*R | GGCTGATATCTGGGTGCCTA |
| *isg15-*F | GAGAGGCAGCGAACTCATCTT |
| *isg15-*R | CCAGCATCTTCACCGTCAGG |
| *cxcl10-*F | GCTCTACTGAGGTGCTATGTTC |
| *cxcl10-*R | GGAGGATGGCAGTGGAAGTC |
| Neg-sense | UUC UCC GAA CGU GUC ACG UTT |
| Neg-anti | ACG UGA CAC GUU CGG AGA ATT |
| ICP27 siRNA1-sense | GCG UGU CGG AGA UCG ACU ATT |
| ICP27 siRNA1-anti | UAG UCG AUC UCC GAC ACG CCG |
| ICP27 siRNA2-sense | GGC GGG UCU CAU UGA AAU ATT |
| ICP27 siRNA2-anti | UAU UUC AAU GAG ACC CGC CAT |

# Supplementary Figures


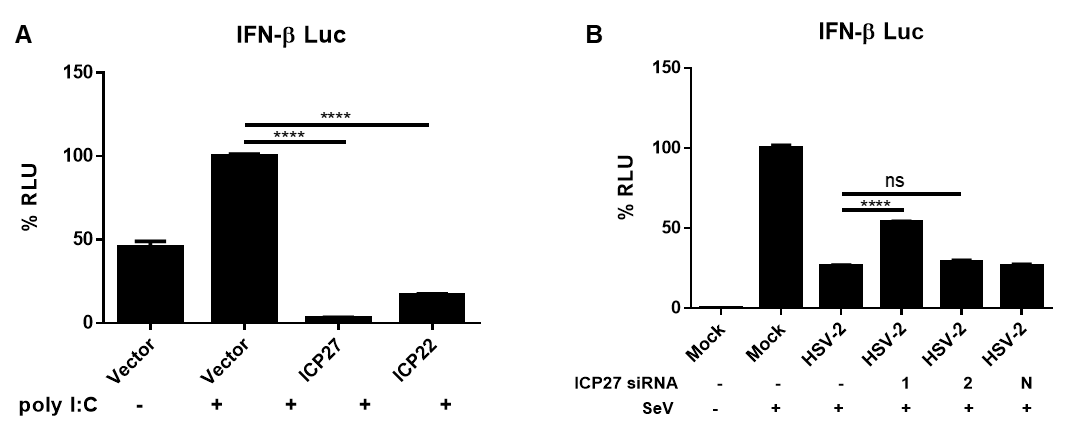


**Supplementary Figure 1.** **HSV-2 ICP27 inhibits IFN-β induction in ME180 cells. (A)** HSV-2 inhibits the Poly(I:C)-induced activation of IFN-β promoter. ME180 cells were seeded in 6-well plates and co-transfected with empty vector pcDNA3.1(+), plasmid expressing HSV-2 ICP27 or ICP22, together with the reporter plasmid p125-Luc and the internal control phRL-TK. At 24 h post-transfection, cells were transfected with 2 µg/well Poly(I:C) or mock-transfected for 16 h, and lysed for DLR assay. Data shown are mean ± SD of three independent experiments. ****P<0.0001. **(B)** Knockdown of ICP27 reduces the capability of HSV-2 in inhibiting IFN-β promoter activation. ME180 cells were seeded in 6-well plates and co-transfected with HSV-2 ICP27 siRNA-1, siRNA-2, or negative control siRNA (N), together with the reporter plasmid p125-Luc and the internal control phRL-TK. At 4 h post-transfection, cells were infected with HSV-2 at an MOI of 0.5 or mock-infected. At 20 h.p.i, cells were stimulated with or without 100 HAU ml^-1^ SeV for 16 h, and lysed for DLR assay. The data shown are representative of three independent experiments, with each condition performed in triplicate (mean ± SD). ****P<0.0001.


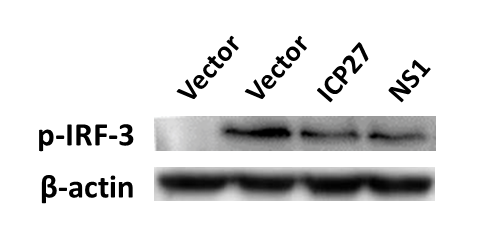


**Supplementary Figure 2. HSV-2 ICP27 inhibits IRF3 phosphorylation.** HEK 293T cells were seeded in 6-well plates and transfected with pcDNA3.1(+), plasmid expressing HSV-2 ICP27 or influenza virus NS1. At 24 h post-transfection, cells were stimulated with or without 100 HAU ml^-1^ SeV for 16 h. Cells were lysed and phosphorylated IRF3 was detected with a p-IRF3 mAb.


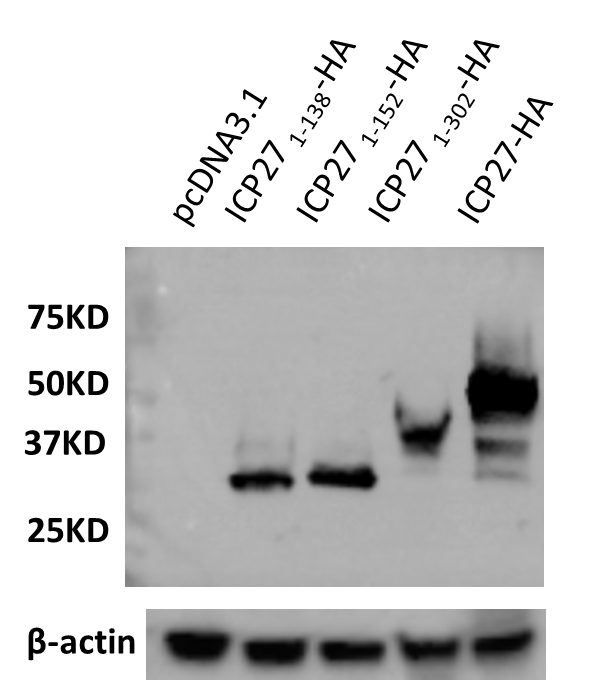


**Supplementary Figure 3.** The expression of HSV-2 ICP27 mutants. HEK 293T cells were transfected with empty vector pcDNA3.1(+), truncated or full-length HSV-2 ICP27 expression plasmid, followed by stimulation with or without 100 HAU ml^-1^ SeV for 16 h, and cells were lysed for Western Blot.
